# Advances in cGAS-STING Signaling Pathway and Diseases

**DOI:** 10.3389/fcell.2022.800393

**Published:** 2022-02-03

**Authors:** Yuting Yang, Yiming Huang, Zhenguo Zeng

**Affiliations:** Department of Intensive Care Unit, First Affiliated Hospital of Nanchang University, Nanchang, China

**Keywords:** cGAS-STING, regulation, innate immunity, inflammation, disease

## Abstract

Pathogens can produce conserved pathogen-associated molecular patterns (PAMPs) after invading the body, which can be specifically recognized by host pattern recognition receptors (PRRs). In recent years, it has been found that cytoplasmic DNA receptors recognize exogenous DNA inducing activation of interferon 1 (IFN1), which is a rapid advance in various research areas. The cyclic GMP–AMP synthase (cGAS) stimulator of interferon gene (STING) signaling pathway is a critical natural immune pathway in cells. Early studies revealed that it plays a crucial regulatory role in pathogen infection and tumor, and it is associated with various human autoimmune diseases. Recently studies have found that activation of cGAS-STING signaling pathway is related to different organ injuries. The present review elaborates on the regulation of the cGAS-STING signaling pathway and its role in various diseases, aiming to provide a theoretical basis for immunotherapy targeting this pathway.

## Introduction

As the most vital human defense system, the immune system exerts strength in safeguarding the body against damage from three aspects—immune defense, immune surveillance, and immune homeostasis. It is generally divided into two branches: one is adaptive immunity, which plays a role by binding B cells or T cell with receptors in a slow but specific manner; and the other is innate immunity, which plays a crucial role in not only preventing pathogen infection as the first line but also activating the adaptive immune response. When the body encounters the stimulation of exogenous microbes or danger signals, pattern recognition receptors (PRRs) encoded by germlines recognize pathogen-associated molecular patterns (PAMPs) and damage-associated molecular patterens (DAMPs), furthermore initiate the process of innate immunity. PRRs were traditionally divided into several categories: Cytosolic DNA sensors, Toll-like receptors (TLRs), NOD-like receptors (NLRs), C-type lectin receptors (CLRs), and RIG-I-like receptors (RLRs) (Pandey et al., 2014). The PAMPs (e.g., microbial nucleic acids, lipoproteins, surface glycoproteins, and membrane components) from pathogens bind to the domains of receptors and transmit the extracellular signal into the cytosol and endosome, resulting in the activation of intracellular signal and regulation of gene expression (Tang et al., 2012). These reactions will activate inflammatory and immune responses (Chen Q et al., 2016). In addition, various endogenous substances (e.g., high mobility group protein B1[HMGB1], S100 proteins, heat shock proteins, hyaluronic acid, ATP, mitochondrial DNA [mtDNA]) released by cells after injury can combine with PRRs and make synergistic effects with PAMP molecules, resulting in further tissue damage.

Over the past few decades, it has become a key factor for innate immunity in mammalian cells that senses microbial pathogens by recognizing their nucleic acids as foreigners, like single-stranded and double-stranded RNAs or DNAs, RNA–DNA hybrids, and cyclic dinucleotides (Janeway, 1989; Mankan et al., 2014; Chen Q et al., 2016; Tan et al., 2018). Even though there are three kinds of sensors identifying intracellular pathogen-derived nucleic acids: TLRs, cytosolic microbial RNA sensors, and cytosolic DNA sensors. The cyclic GMP-AMP synthase (cGAS) can efficiently interact with cytosolic DNA, leading to activating downstream factors (Tan et al., 2018). Most recently, the cGAS-STING signaling pathway has been extensively investigated in various pathological settings. The function of the cGAS-STING signaling on cancers has been reviewed in many literatures (Bose, 2017; Ng et al., 2018; Li A et al., 2019). In the present review, we mainly focus on reviewing the functional roles of the cGAS-STING pathway in diseases and different organ injuries. We also discuss how to activate cytoplasmic cGAS receptors and detail the numerous regulatory mechanisms that control this pathway.

## cGAS-STING Signaling Pathway

### cGAS

**FIGURE 1:**
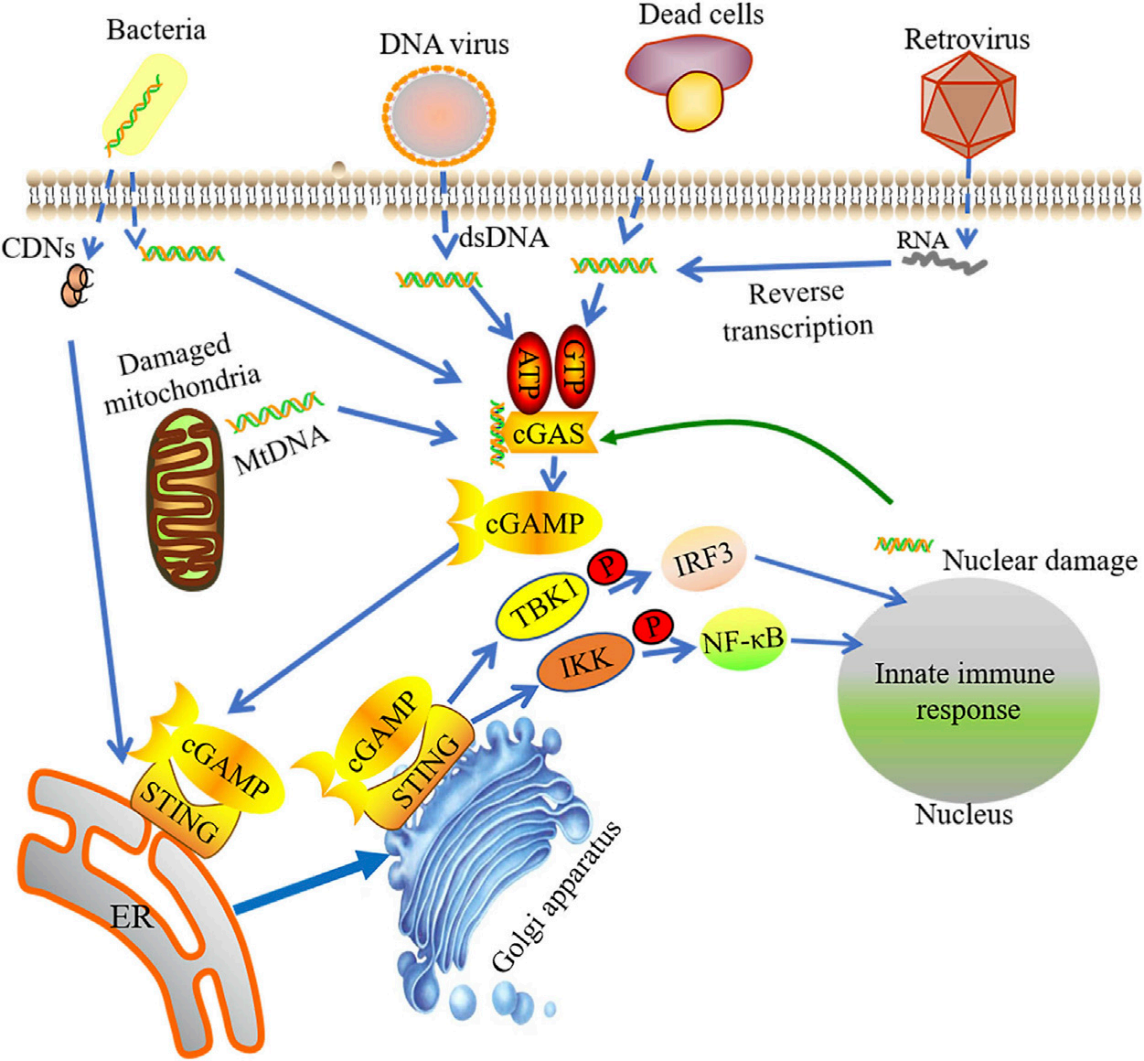
The activation of cGAS-STING signaling pathway. Foreign DNA and RNA in cytoplasm could be sensed by cGAS, which sub-sequently facilitates the synthesis of cyclic GMP-AMP (cGAMP) by binding with GTP and ATP. The integration of cGAMP and STING induces STING activation. STING is then switched from the endoplasmic reticulum (ER) to perinuclear endosomes assisted by the Golgi apparatus. STING signaling can sense and response to foreign DNA and RNA in cytoplasm. The STIGN-TBK1 compound triggers innate immune response via phosphorylating IRF3 or NF-kB.

cGAS-STING signaling can sense and respond to foreign DNA and RNA in cytoplasm (Figure 1). cGAS is a STING-dependent cytoplasmic DNA receptor and a well-documented example of PRR, which recognizes and responds to pathogen DNA and RNA as well as self-DNA. Previous study supports that cGAS exists only in the cytoplasm, thus separated with self-DNA located in the nucleus and mitochondria, but now more evidence shows that cGAS also appears in the nucleus and is closely integrated with the nucleosome. Nuclei cGAS has high binding affinity with the nucleosome, which inhibits cGAS binding to double-stranded DNA (dsDNA) to maintain it in an inactive format (Gentili et al., 2019; Volkman et al., 2019; Zhao et al., 2020). Through electrostatic interaction, the positive-charged cGAS combined with the sugar phosphate skeleton on DNA to form hydrogen bonds (Civril et al., 2013). cGAS can sense the amino-terminal domain of dsDNA, but not the DNA sequence, which facilitates the synthesis of cyclic GMP-AMP (cGAMP) by GTP and ATP (Sun et al., 2013; Chen Q et al., 2016). Recently studies have reported that single-stranded DNA (ssDNA) forms a specific Y-type and activates cGAS with an unknown activation mechanism (Chen Q et al., 2016). A recent report demonstrated that mitochondrial transcription factor A (TFAM) and HMGB1/2 have abilities to adjust the structure of DNA in advance, benefiting from forming DNA U turns and enhancing the effective DNA binding with cGAS. TFAM is located in the mitochondria and can be liberated into the cytosol when mitochondrial stress occurs, which promotes cGAS to identify mtDNA. HMGB1 protein can move back and forth between the nucleus and the cytoplasm, promoting the identification of cytoplasmic DNA (Andreeva et al., 2017).

There is a highly conserved activation loop in cGAS. The activation loop can insert into the first big groove of B-DNA and the prominent outer loop then moves inward, leading to a conformational change and the formation of a new positive patch to allow the next DNA binding. The activation loop is constantly moved inward to rearrange the active sites to catalyze the formation of cGAMP. Although both dsRNA and A-DNA can bind cGAS, they cannot push the activation loop inward, which results in cGAS and subsequent reactions cannot be activated (Andreeva et al., 2017).

cGAS has a catalytic domain for ATP and GTP binding (Kuchta et al., 2009) and a unique “zinc-thumb” structure site for B-DNA binding (Pandey et al., 2014). cGAS binds with DNA in a homologous dimerization and forms a 2:2 complex that changes its conformation. Catalytic domain in cGAS binds with ATP and GTP and catalyzes the production of cGAMP (Cai et al., 2014). Although cGAS dimerization may occur without cytoplasmic DNA, its DNA binding capacities rely on the target DNA length. cGAS efficiently recognizes dsDNA with a length greater than or equal to 36 bp along with downstream STING activation (Kumar, 2019). As a unique endogenous synthesis method in mammals, cGAMP synthesized by cGAS is named as atypical cyclic dinucleotides (CDNs) owing to its two unique phosphodiester bonds, which can activate STING (Pandey et al., 2014).

### STING

DNA binding induces conformational changes in cGAS with catalyzing cGAMP production. cGAMP, as a second messenger, recruits its downstream effector protein STING, a membrane-spanning protein. STING is composed of four transmembrane domains (TM1∼4) and cytoplasmic c-terminal domain (CTD) structure. Shang et al. show that a dimeric format can be formed through the interaction of the transmembrane and cytoplasmic regions of STING. Ligand-binding domain of STING can be closed in the presence of cGAMP, resulting in the structural changes of the ligand-binding domain. The conformational change will produce the STING tetramer (Shang et al., 2019), which induces STING activation. Subsequently, STING is switched from the endoplasmic reticulum (ER) to perinuclear endosomes assisted by the Golgi apparatus. A complex can be formed by STING and TANK-binding kinase 1 (TBK1). TBK1 has capacity to phosphorylate various transcription factors, such as nuclear factor κ-light-chain-enhancer of activated B cells (NF-kB) and interferon regulatory transcription factor 3 (IRF3). The transcription of innate immunity-related genes, like type I IFNs, can be initiated by the formation of STING-TBK1 complex (Ishikawa and Barber, 2008; Saitoh et al., 2009; Tanaka and Chen, 2012; Mukai et al., 2016). Therefore, activation of cGAS-STING signaling pathway induces upregulation of inflammatory genes and turns on the downstream signaling.

## Regulation of cGAS-STING Signaling Pathway

### Regulation of DNA Level

The main source of spontaneous DNA double strand rupture is derived from DNA replication in proliferating cells. Factors affecting the replication process may cause abnormal DNA damage response and cytoplasmic DNA accumulation. Kreienkamp et al. demonstrated that progesterone, a critical factor in progeria, participates in the replication stress process through generating replication fork disruption and nuclease-mediated degradation in the stalled forks. The process is the main sources of DNA damage in HGPS animal models and cells (Kreienkamp et al., 2018).

It is well known that BRCA2 tumor suppressor takes part in promoting DNA replication and DNA double-strand break repair. Recent studies have shown that BRCA2 mutation caused sustained DNA damage and abnormal chromosome separation by creating replication fork instability and nucleolysis degradation, thereby activating cGAS-dependent innate immune responses (Zimmer et al., 2016; Lai et al., 2017; Reisländer et al., 2019). Reisländer’s group has found that there are dual responses to BRCA2 depletion. The early acute response is composed of downregulation of genes, which are related to the cell cycle, DNA replication, and repair, along with cell cycle arrest in G1. However, the late chronic response is predominantly consisted of upregulation of interferon-stimulated genes (Reisländer et al., 2019).

TREXl, an Aicardi syndrome gene, mainly codes for mammalian 3 ′→5′ DNA nucleic acid exonuclease, whose function is to degrade or unchain DNA. It has been reported that TREX1 expression is required to prevent DNA accumulation in the dead cells, which might cause an autoimmune response (Tao et al., 2019). DNase2 is an endonuclease mainly located in the phagolysosomal compartment that degrades excess DNA in lysosomes. DNase2 and TREX1 (DNase3) target dsDNA and ssDNA for degradation in normal healthy cells, respectively, which prevents free DNA accumulation in cytosol (Stetson et al., 2008; Geijtenbeek, 2010; Lan et al., 2014). In the case of enzyme dysfunction or decline, or DNA specific resistance, abnormal DNA accumulates in the cytoplasm, resulting in excessive activation of the cGAS-STING pathway. It has been found that TFAM is a crucial protein to maintain mtDNA functional stability (Campbell et al., 2012). A recent study has found that the deletion of TFAM caused the abnormal mtDNA accumulation in the cytosol (West et al., 2015; Chung et al., 2019). Fat specificity of disulfide bond sample—a redox enzyme protein disulfide-bond-A oxidoreductase-like protein (DsbA-L)—was initially identified as a partner in the mitochondrial protein amyloid. Few studies have proved that DsbA-L is a key to maintain the integrity and stable mitochondria function. Lacking DsbA-L leads to mitochondrial dysfunction and mtDNA release, which activates cGAS-cGAMP-STING pathway and triggers inflammatory responses (Bai et al., 2017). Barrier-to-autointegration factor 1 (Banf1) has abilities to recognize exogenous DNA and inhibit chromosomal integration (Ma et al., 2020) or genome replication (Ibrahim et al., 2011). Ma et al. showed that Banf1 could modulate basal cell-intrinsic immunity using a CRISPR-Cas9 screening strategy. Mutation of Banf1 leads to a large amount of cytosolic accumulation of dsDNA, which induces IFN-stimulated gene expression through cGAS-STING-IRF3 signaling pathway activation (Ma et al., 2020). Collectively, abnormal levels of DNA in nuclei and cytoplasm will result in activation of cGAS-STING signaling under various pathological conditions.

### Regulations of cGAS

Previous studies proved that cGAS is predominantly located in the cytoplasm. However, there is now new evidence showing that cGAS can also be in the nucleus and bind to chromatin under certain conditions. Yang et al. labeled cGAS with GFP in fibroblast cells (MEF) from cGAS mutant mice and tested cells in different growth densities by immunofluorescence. Surprisingly, they found that cGAS was associated with DNA and mainly existed in the nucleus in the rapidly dividing cells. In contrast, cGAS primarily existed in cytosol in the nondividing cells (Yang et al., 2017). Recent studies confirm that the endogenous inactivated cGAS is a nucleoprotein which closely connects with the nuclear membrane by a salt-resistant interaction no matter what cell cycle phase or activation states. NTase domain of cGAS and intact nuclear chromatin are essential to maintain tight nuclear tethering. This mechanism differs from the activated cGAS recognizing abnormal DNA within cytoplasm, restrained cGAS cannot play its catalytic activity (Volkman et al., 2019).

It is found that the regulation of cGAS is mainly through the post-translational modification in its enzyme activity and protein expression (Chen Q et al., 2016; Motwani et al., 2019). The types of post-translational modification include phosphorylation, acetylation, monoglutamylation, polyglutamylation, ubiquitination, and SUMOylation. For example, phosphorylation of the kinase Akt26 at Ser305 or Ser291 sites of cGAS and glutamylation of the enzymes TTLL4 at glu302 and TTLL6 at glu272 inhibit cGAS activity, while glutamylation of carboxypeptidases CCP5 and CCP6 activates cGAS. ER ubiquitin ligase ring finger protein 185 (RNF185) enhances cGAS’s catalytic ability (Wang et al., 2017). It is known that the tripartite motif-containing protein 38 (TRIM38), an E3 ubiquitin ligase, is able to inhibit innate immune and inflammatory responses induced by viral RNA, lipopolysaccharide (LPS), and tumor necrosis factor α (TNF-α) (Hu et al., 2014; Hu et al., 2015). In addition, Hu et al. reported that TRIM38 can catalyze SUMOylation of murine cGAS at K217 to enhance the stability of cGAS in the early stage of viral exposure to prevent cGAS degradation. TRIM38 induces K48-linked polyubiquitination of cGAS at K464 in the late stage of infection to promote the innate immune response. cGAS is desumoylated by SUMO-specific protease2 and subsequently degraded via proteasome. cGAS can be cleaved by inflammation and apoptotic caspases. cGAS activation is limited by gasdermin D (GASMD) (Hu et al., 2016). Thus, the activity of cGAS is regulated by many factors.

AIM2 inflammasome, a PRR that recognizes intracellular or cytoplasmic dsDNA, is induced by IFN and belongs to the hin-200 (ifi-200/hin-200) protein family. The AIM2 complex is composed of AIM2, apoptosis-associated speck protein, and caspase-1. Several studies have shown that AIM2 inflammasome inhibits endogenous cGAMP production by promoting caspase-1-dependent cell death (Corrales et al., 2016; Kumar, 2019).

Autophagy can transport cytoplasmic substrates into the lysosomes to form autophagosome, which is a double membrane-bound vesicle. The autophagy procedure often attends the protective immune response in homeostasis. Beclin1 (BECN1), an autophagy protein, inhibits cGAMP synthesis through interacting with cGAS, thereby halting the production of type 1 IFN when exposed to cytoplasmic dsDNA or HSV1 infection. The presence of BECN1 protein can increase autophagy-mediated cytoplasmic DNA degradation by inhibiting cGAS-STING signaling pathway (Ishikawa and Barber, 2008). Increasing autophagic activity through p62 upregulation causes cGAS degradation by K48-linked cGAS ubiquitination. TRIM14 expression, an interferon-activated gene, was increased during DNA virus infection. Deubiquitinating enzyme USP14, recruited by TRIM14, can degrade cGAS (Chen M et al., 2016).

G3BP1 participates in a formation of stress particles which are composed of a variety of proteins and mRNAs. G3BP1 has a nucleic acid helicase activity that can partly dissociate double-stranded DNA, double-stranded RNA, and DNA:RNA hybridization chain. In recent years, it has been identified that G3BP1 is a cGAS auxiliary protein. G3BP1 not only promotes the combination of cGAS and DNA, but also directly affects the enzyme activity of cGAS (Liu ZS et al., 2019). Mutation of G3BP1 blocks intracellular cGAS condensation and reduces cGAMP synthesis induced by DNA stimulation, which will inhibit interferon responses to intracellular DNA and viral infection (Liu ZS et al., 2019). Another protein having synergistic effect during cGAS sensing DNA is CCHC-type zinc-finger protein ZCCHC3. *In vitro/vivo* experiments have proved that ZCCHC3-deficiency negatively affects cGAS recognizing cytoplasmic abnormal DNA, thus impairing the subsequent inflammation. In addition to directly interacting with cGAS to modulate cGAS oligomerization, ZCCHC3 itself can also combine with a variety of dsDNA (B-DNA, Z-DNA, and virus-DNA) but not ssDNA to promote cGAS activation (Lian et al., 2018).

### Regulations of cGAMP

cGAS, ATP, GTP, and DNA are indispensable in the process of cGAMP production catalyzed by cGAS (Civril et al., 2013; Sun et al., 2013; Kato et al., 2017). Two special phosphate bonds, the 2 ′,5′ phosphate bond and the 3 ′,5′ phosphate bond, are contained in the cGAMP. Both phosphate bonds are linked by the 3 ′,5′ phosphate bond (Ablasser et al., 2013). The 2 ′,5′cGAMP is a strong ligand of human STING, which produces a higher level of INF-β (Ablasser et al., 2013; Li et al., 2013).

Phosphodiesterase, such as ecto-nucleotide pyrophosphatase/phosphodiesterase family member 1 (ENPP1), inhibits bone mineralization. Phosphodiesterase can also hydrolyze extracellular ATP in mineralizing cells. It has been found that ENPP1 can hydrolyze the 2′,5′ phosphodiester bond and 3′,5′ phosphodiester bond of 2′,3′ cGAMP in turn to reduce the level of cGAMP (Li et al., 2014; Kato et al., 2018) and inhibit cGAS-STING activation.

### Regulations of STING

STING, a nucleic acid receptor adaptation protein, acts on multiple intracellular signaling pathways. Except cGAS, other cytoplasmic receptors, such as DDX41 and IFI16, have abilities to sense DNA or CDNs and activate STING (Beutler et al., 2006; Parvatiyar et al., 2012).

Various post-translational modifications are important regulatory approaches to maintain the stability of the STING protein (Motwani et al., 2019; Lee et al., 2020). The phosphorylation and ubiquitination of STING are important for the regulation of its activity. TBK1 leads to phosphorylation of STING at Ser358 and 366, which activates STING (Zhong et al., 2008). STING can be targeted by the ER-associated E3 ubiquitin ligase RING finger protein, which is involved in K48-linked poly-ubiquitination at K150 and proteasome-mediated degradation of cGAS (Zhong et al., 2009). Other E3 ubiquitin ligases, such as TRIM29 and TRIM30α, promote STING degradation by poly-ubiquitination (Wang et al., 2015; Li et al., 2018). Both insulin-induced gene 1 protein and E3 ubiquitin ligase complex of AMFR can recruit TBK1, leading to polyubiquitination and degradation of STING (Wang et al., 2014). Dimerization of STING can be enhanced by ZDHHC1, an ER-associated palmitoyltransferase (Zhou et al., 2014). STING interacts with sterol regulatory element-binding protein cleavage-activating protein (SCAP), facilitating IRF3 recruitment. Additionally, K48-linked ubiquitin chains can be removed by the deubiquitinase enzyme CYLD to stabilize STING protein (Chen W et al., 2016). A recent study has found that SUMOylation of STING at K337 induced by TRIM38 can impede STING degradation. The modification facilitates STING activation and IRF3 recruitment (Hu et al., 2016).

During viral infection, nitro fatty acids can covalently modify STING through nitroalkylation and further inhibit STING palmitoylation (Hansen et al., 2018). Immediate-early 2 gene product (IE86) promotes STING degradation by using its amino acid position 136–289, which significantly reduced the STING-induced IFN-promoter activity (Zhong et al., 2009). Under the activation of cGAMP, STING is transported from ER to the position of the parietal/Golgi body and endoplasmic sites, and act as a scaffold protein to recruit TBK1 and IRF3. NLR family CARD-containing 3 (NLRC3) inflammatory protein inhibits STING signaling by directly binding its CARD domain with STING and TBK1. This interaction involves the C-terminal soluble tail and residues 81–111 of STING and the N terminus of TBK1, which have the kinase-domain (Zhang et al., 2014; Nascimento et al., 2019).

## The Function of cGAS-STING Pathway on Diseases

### The Function of cGAS-STING Pathway in Acute Injury

It is well known that neutrophils, the first line of defense against foreign pathogen invasion, play a vital role in the innate immune system (Silvestre-Roig et al., 2019). In addition to phagocytosis of pathogenic microorganisms, production and secretion of granular antibacterial molecules can also produce neutrophil extracellular traps (NETs) under inflammatory conditions (Castanheira and Kubes, 2019). NETs is a network of fibers with nuclear or mtDNA as the skeleton, decorated with spherical protein domains of different sizes. Among them, the fibrous network structure is the main functional substance of NETs, including deconcentrated chromatin and histone, antimicrobial peptides, and cell-specific protease (Allam et al., 2014; Nascimento et al., 2019). NETs is a double-edged sword for the host. Although NETs can play a defensive role in the inflammatory state by capturing and killing microbes (Bruns et al., 2010; McDonald et al., 2012), excessive NETs can aggravate the acute (Tanaka et al., 2014; Jiménez-Alcázar et al., 2017) or chronic injury of the organization (Grabcanovic-Musija et al., 2015; Sollberger et al., 2018). In addition to various pathogens, sterile inflammation is not a neglectable source of NETs (Wong and Wagner, 2018; Sorvillo et al., 2019).

Early studies thought that the nucleic acid of NETs may come from the nucleus (Brinkmann et al., 2004). Researchers are more inclined to support that the release of nuclear DNA (nDNA) is a result of neutrophils necrosis, which did not participate in the composition of NETs. mtDNA is indeed involved in the process of NETs formation (Yousefi et al., 2009; Lood et al., 2016; Yousefi and Simon, 2016). Besides, mtDNA also plays a central role in the induction of NETs, especially in trauma and surgery settings (McIlroy et al., 2014; Itagaki et al., 2015). Lood et al. stimulated normal neutrophils with PMA or ribonucleoprotein-containing immune complexes (RNP ICs). They confirmed that the NETs were mainly composed of oxidized mitochondrial DNA. The induction of NETs was dependent on cGAS or STING (Lood et al., 2016). The phenomenon of mtDNA and oxidized mtDNA-inducd NETs formation was also discovered in tissue trauma and injury models established by Lui et al.

**FIGURE 2:**
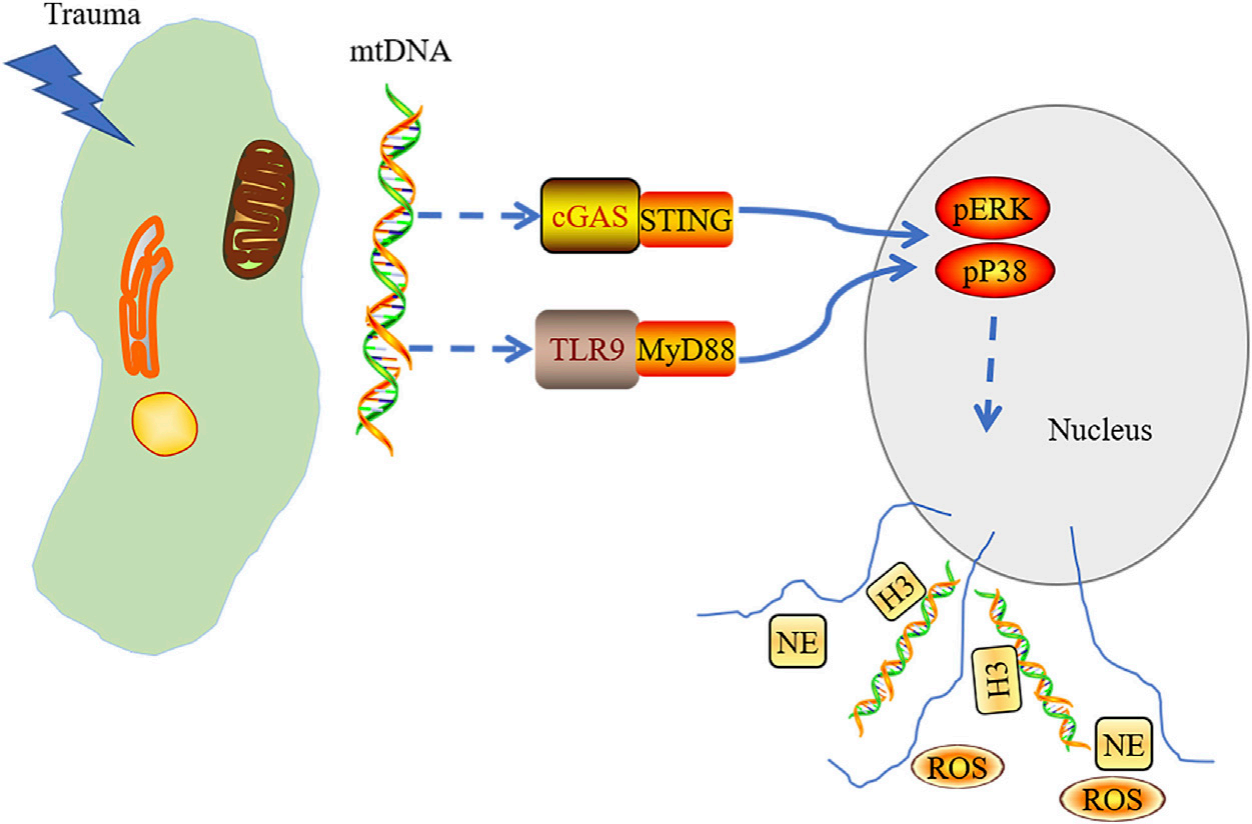
mtDNA released into cytoplasm in acute tissue injury induced by trauma and triggered the activation of cGAS-STING and TLR9-MyD88. Stress signaling was transferred into the nucleus through activated cGAS-STING and TLR9-MyD88 pathways, which mediate the activation of the p38 MAPK and ERK1/2 signaling and the formation of neutrophil extracellular trap.

By intravenous injection of mitochondrial DNA into mice, the generation of NETs was weakened in the lungs of STING^−/-^ mice and TLR9^−/−^ mice. Extracellular regulated protein kinases (ERK)1/2 and p38 mitogen-activated protein kinase (MAPK) inhibitors could significantly reduce NETs-associated protein expression. Taken together, we conclude that mtDNA induces NET formation, which is mediated by the activation of STING, TLR9, the ERK1/2, and p38 MAPK during injury and trauma, subsequently inducing aseptic inflammation (Liu L et al., 2019) (Figure 2). The degradation of NETs relies on DNase I and macrophages (Farrera and Fadeel, 2013). Targeting inhibition of STING or TLR9 might alleviate the NETs-induced inflammation. Thus, the formation of the NETs is the key to cause tissue damage, indicating that adjusting the level of the NETs may be the target in the clinical treatment of tissue trauma.

In the ischemic stroke mice model, the NETs induced by activation of cGAS-STING and the subsequent type 1 interferon response contribute to tissue plasminogen activator (tPA)-associated hemorrhage, resulting in increased loss of cerebrovascular integrity and amplified blood–brain barrier damage. Ranran Wang et al. discovered that the treatment with DNase I or PAD4 deficiency could restore cerebrovascular complications of tPA from two patterns: directly reducing the level of the NETs or indirectly regulating NETs via reversed tPA-mediated upregulation of cGAS (Wang et al., 2021). Therefore, inhibition of NETs or cGAS will provide a new avenue to block tPA thrombolysis upon ischemic stroke pathological condition.

### The Function of cGAS-STING Pathway in Pneumonopathy

Chronic obstructive pulmonary disease (COPD) is a severe chronic inflammatory disease. COPD causes lung functional loss with chronic lung inflammation, which leads to lung tissue destruction. This inflammation involves multiple innate and adaptive immune responses, like activation of epithelial cells and inflammatory cells (e.g., macrophages, neutrophils, lymphocytes, dendritic cells), release of varieties of inflammatory mediators (e.g., lipid, cytokines, NLRP3 inflammasomes), and oxidative stress which seems like a major driving factor (Barnes, 2016). The production of reactive oxygen species (ROS) under oxidative stress leads to activation of phosphoinositide 3-kinase, NF-kB (Kafoury and Kelley, 2005), and histone acetyltransferase activity (Tomita et al., 2003). ROS increases mitochondrial damage with overproduction of ROS in lungs (Wiegman et al., 2015). The vicious cycle causes numerous abnormal nuclear and mtDNA release to cytoplasm (Juan et al., 2021).

**FIGURE 3:**
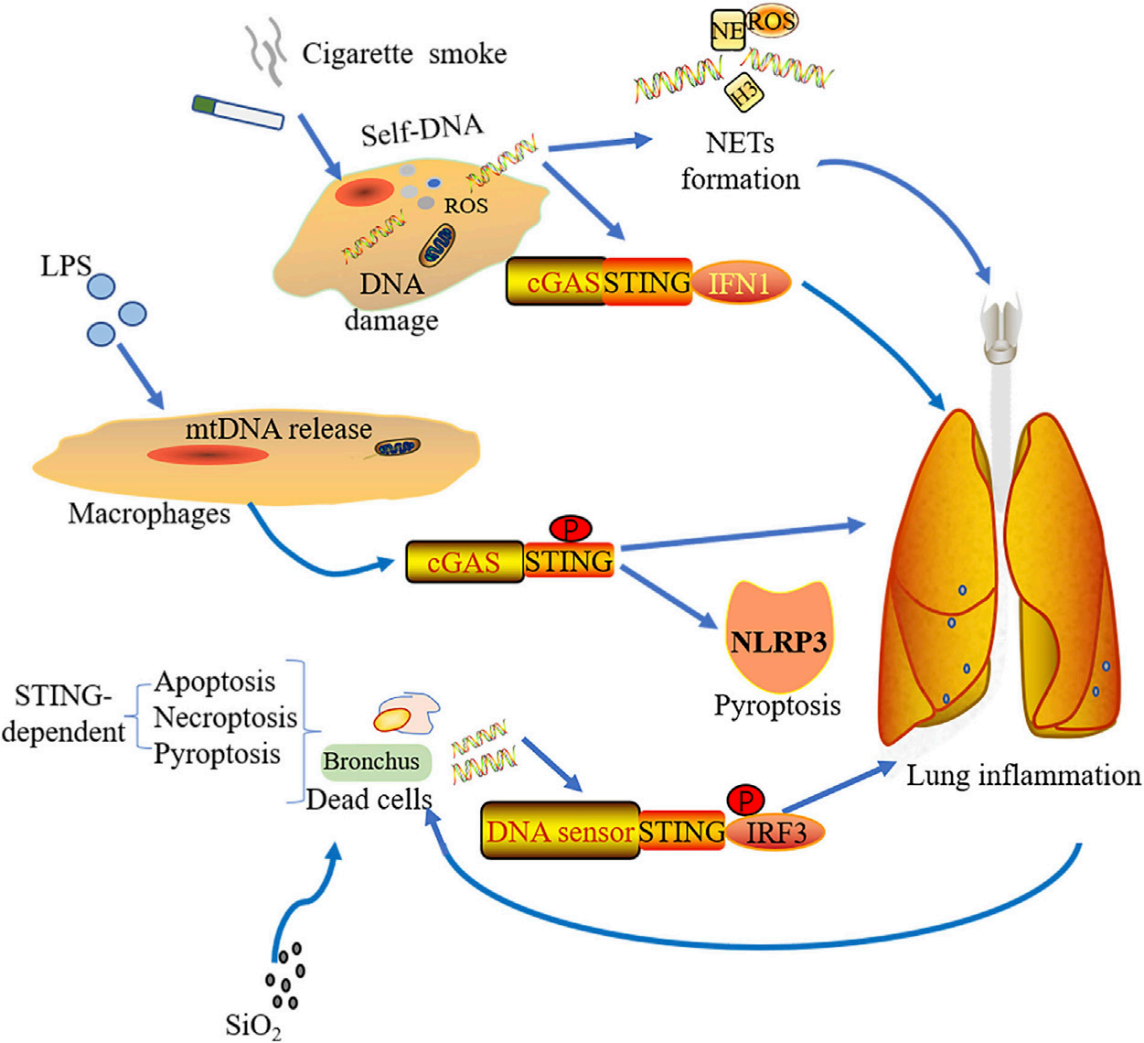
Stimulations from various sources induce pulmonary inflammation through the cGAS-STING pathway. Cigarette smoke mediates lung inflammation by the activation of cGAS-STING-IFN1 and the formation of NETs. With simulations of LPS, macrophage could release mtDNA, which was sensed by cGAS-STING and induced NLRP3-mediated pyroptosis as well as lung inflammation. Silica exposure to bronchus leads to different forms of STING-dependent cell death including apoptosis, necroptosis, and pyroptosis. The dsDNA from the dead cells triggers lung inflammation via STING-IRF3 signaling.

Smoking, as a main cause of COPD, also induces DNA damage and cell death with lung inflammation which mainly depends on ROS production (Loukides et al., 2011; Kirkham and Barnes, 2013; Barnes, 2016). Many signaling pathways refer to smoking-induced COPD, including Wingless/integrase-1 (WNT) signaling (Heijink et al., 2016), PINK (PTEN-induce putative kinase1)-PARK2 pathway (Ito et al., 2015), and NF-kB signaling (Yuan et al., 2018). Nascimento et al. discovered that cGAS-STING signaling also contributed to the mice COPD model under acute cigarette smoke (CS) exposure. dsDNA within broncho-alveolar space, caused by different mechanisms (damaged cells, defective repair of DNA damage, or ROS), promotes NETs formation and can be sensed by cGAS but not TLR9, inducing type I IFNs expression (Figure 3). By using genetic knockout mice, it was found that lung inflammation was alleviated in the absence of cGAS or STING, which means that the pathway might be a potential therapeutic target for controlling lung inflammation resulting from cigarette smoking (Nascimento et al., 2019).

In CS-induced and adenovirus-associated COPD acute exacerbation (AECOPD) mice experiments, recombinant adenovirus vectors (rAdVs) could activate STING-IFN-β pathway by sensing DNA of Ads. However, the expression of STING was significantly reduced 12 weeks after CS exposure. The activation of STING-IFN-β pathway exerts the protective effects via promoting clearance of rAdVs DNA while STING deficiency leads to amplifying inflammation and aggravating alveolar destruction (Qin et al., 2019). Among bacterial infection-induced by AECOPD, the most common bacteria is nontypeable *Hemophilus* influenzae (NTHI) (Sethi and Murphy, 2008). Previous work has confirmed the effects of TLR4 (Pang et al., 2008) or NF-κB signaling pathway (Shuto et al., 2001) in NTHI infection. Lu, Zhang et al. established a mimic COPD murine model via intrabronchial infection of LPS for a period of 4 weeks. They discovered that NTHI DNA could upregulate STING expression, which triggers I-IFN responses in a TBK1-dependent manner. Additionally, the overproduction of STING-related cytokines largely depends on cGAS activation under NTHI and NTHI DNA stimulation (Lu et al., 2018). The diverse effects of STING in different periods of COPD deserve further investigation to explore the therapeutic application.

Silica exposure is known to cause lung inflammation and is an important cause of interstitial lung disease (ILD) (Leung et al., 2012). Through the analysis of pneumonectomy samples from ILD patients with lung transplantation, Benmerzoug et al. showed that dsDNA and CXCL levels were increased in the sputum of silicosis patients and ILD patients with cGAS-STING-IRF1 activation. Mice exposed to silica showed that release of nDNA and mtDNA result from different forms of cell death including apoptosis, pyroptosis, and necroptosis. Subsequently, this endogenous dsDNA was sensed by cGAS resulting in activated STING-mediated type I IFN response. It is worth observing that whether early or late phases of silica exposure, STING drives lung inflammation, whereas the sensing effect of cGAS was not prominent early after silica exposure. Silica exposure not only promotes the development of lung inflammation by cGAS-STING-IRF1, but also induces inflammatory cell death depending on cGAS/STING signaling (Figure 3). Interestingly, they also found that different DNA sensors activated STING in dendritic cells and macrophages. Dendritic cells rely on cGAS to activate STING, while macrophages rely on other DNA sensors (Benmerzoug et al., 2018).

In the clinic, thermal trauma is one of the common risk factors of severe lung dysfunction. Deep skin damage often is accompanied by extensive cell death, which releases pro-inflammatory DAMPs including mtDNA (Liu R et al., 2017; Liu R et al., 2019). Additionally, in mice subjected to burn injury, mtDNA damage appeared in the lungs at the earliest and had the most serious effect. The mitochondrial dysfunction induced by thermal injury was marked time-dependent (Szczesny et al., 2015). Comish and his colleagues demonstrated that plasma mtDNA was increased after thermal trauma in burn-induced acute lung injury (ALI) of rats. mtDNA in lung tissues was reduced 24–72 h following injury, which may be because of sustained mitochondrial DNA damage within the lung and oxidative cell damage (Comish et al., 2021). Given that TLR9 expression is downregulated after burns (Shen et al., 2012), they demonstrated that the cGAS-STING pathway activation is involved in burn-induced ALI. However, the pathological role of the pathway is still unclear.

The cGAS-STING pathway not only plays a role in non-bacterial inflammation but also has effects on LPS-induced ALI. Cytosolic LPS could induce mtDNA release into the cytosol in endothelial cells, which is mediated by mitochondrial pores with activating the pore forming protein GASDMD, resulting in impaired endothelial cell proliferation and aggravated inflammatory injury by downregulating cGAS-YAP1 signaling. The regenerative ability of endothelial cells was restored when cGAS was deficient in mice under acute inflammatory lung injury (Huang et al., 2020). mtDNA can be recognized by cGAS under LPS stimulation, enhancing STING phosphorylation in macrophages. This will subsequently trigger NLRP3-mediated pyroptosis (Figure 3). LPS could raise the macrophagic pyroptosis by means of heightening the transcriptional activation of STING via c-Myc (Ning et al., 2020). Therefore, the cGAS-STING pathway-mediated inflammation plays a crucial role in ALI.

### The Function of cGAS-STING Pathway in Kidney Disease

It is well accepted that the kidney is an important organ for maintaining water, electrolyte and acid-base balance, which is mainly achieved by 99% of glomerular reabsorption. Therefore, mitochondria, which provides huge energy for this energy-consuming process, plays an important role in renal homeostasis (Ishimoto and Inagi, 2016). Ischemia-reperfusion injury, sepsis, and nephrotoxins can be observed in acute kidney injury (AKI). A total of 7–25% of AKI are derived from drug-induced adverse effects (Nash et al., 2002; Bentley et al., 2010) and are featured with excessive inflammation and tubular injury (Mulay et al., 2016).

**FIGURE 4:**
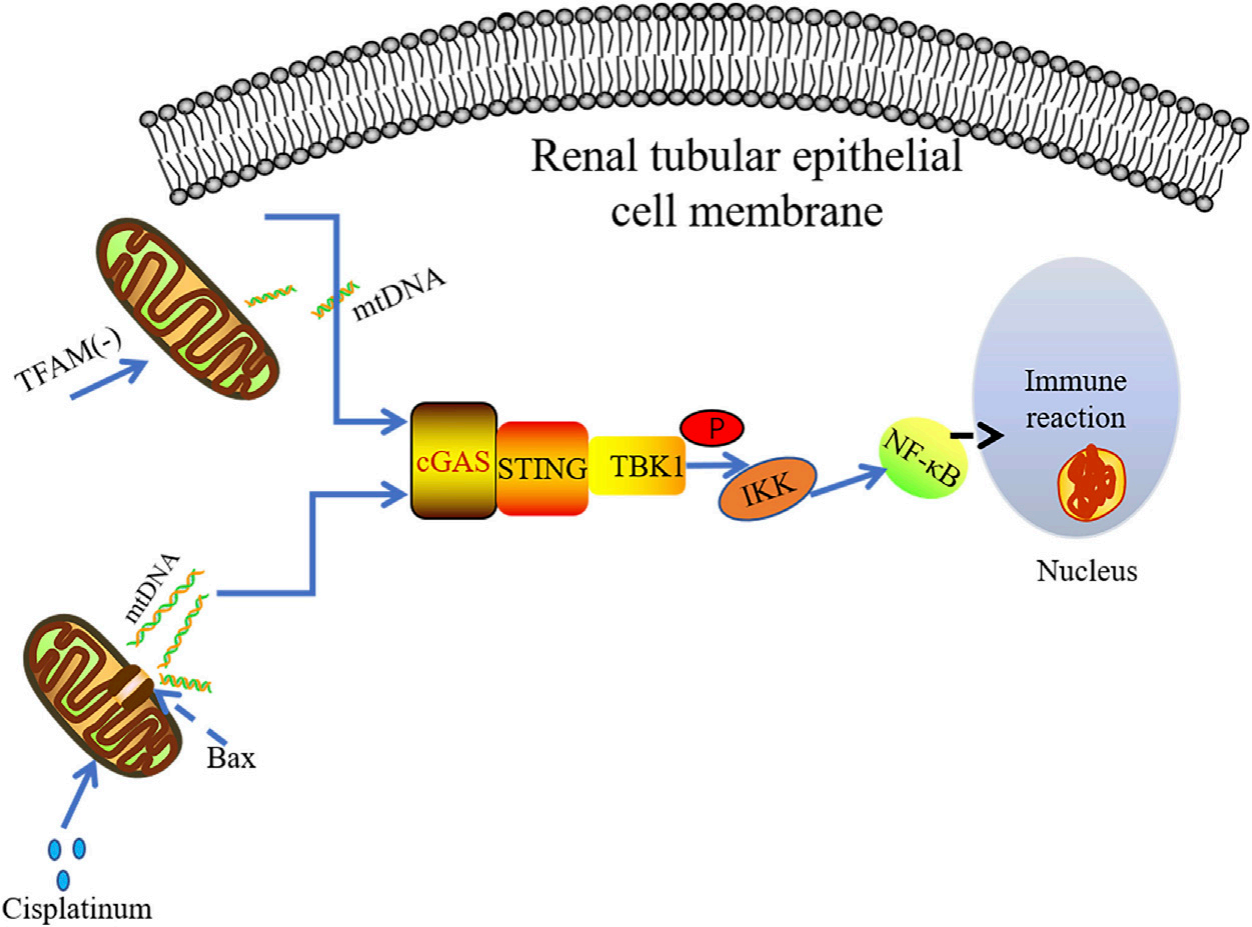
mtDNA leakage plays a key role in cisplatin-induced kidney inflammation. The damaged mitochondria can result in cGAS-STINF-NF-kB pathway activation in the renal tubules. The absence of TFAM leads to mitochondrial damage and the release of mtDNA, which mainly activates the cGAS-STING-NF-κB pathway to induce inflammatory response.

It has been found that the use of cisplatin can cause dose-dependent renal toxicity in patients with malignant tumors. The induction of renal toxicity is closely related to mitochondrial damage and inflammation (Pabla and Dong, 2008; Miller et al., 2010). TLR9 can recognize mtDNA, contributing to AKI (Tsuji et al., 2016). Maekawa et al. conducted immunohistochemical analysis on kidney specimens of non-AKI patients and AKI patients, finding that STING and P65 were highly expressed in the kidney of AKI patients. It was observed that mtDNA leaking into the cytoplasm of renal tubule cells in the cisplatin-induced mouse AKI model. Cytoplasmic DNA was released from mitochondria through the macropore BCL-2-like protein 4(BAX) (McArthur et al., 2018; Maekawa et al., 2019). The BAK/BAX macropores have been confirmed to allow mtDNA to be exposed to the cytoplasm by making the herniated inner membrane an outlet (McArthur et al., 2018). Subsequently, the abnormal mtDNA in cytosol could be sensed by cGAS, resulting in STING-dependent inflammation and renal injury. However, only TBK1 and p65 phosphorylation were found in primary culture renal proximal tubular epithelial cells treated with cisplatin without IRF3 phosphorylation. These data indicate that cisplatin-induced renal inflammation may activate non-classical cGAS-STING signaling pathway in renal tubule cells (Figure 4). The tubular inflammation induced by cisplatin was significantly reduced under the circumstance of STING gene mutation or application of STING inhibitor C-176 (Maekawa et al., 2019), further indicating there is the pro-inflammatory effect of the cGAS-STING pathway in AKI.

Chronic kidney disease (CKD) is also a serious worldwide health problem, and atherosclerosis is a prominent mortality risk factor for patients with CKD (Jha et al., 2013). Vascular smooth muscle cells (VSMCs) are known to have vital effects in sustaining plaque stability of atherosclerosis. In the CKD mice model with polipoprotein E-deficient (ApoE^−/−^), it was observed that VSMCs could sense mitochondrial damage resulting from oxidative stress via the activation of the cGAS-STING pathway. The subsequently triggered IFN-I response facilitates plaque vulnerability on account of inducing VSMCs premature senescence and phenotypic switching. In contrast, pretreatment of hVSMCs with H-151(STING inhibitors) or ruxolitinib (JAK-STAT inhibitor) significantly alleviates IFN-I response, premature senescence, and phenotypic switching in VSMCs induced by CKD serum (Bi et al., 2021).

It has been known that depletion of TFAM can lead to severe mitochondrial defects, which is an important way to release mtDNA into the cytoplasm (West et al., 2015; Chung et al., 2019). Chung et al. demonstrated that TFAM was lost in renal tubular cells derived from patients with fibrosis and animal models. Their data indicate that the loss of TFAM directly activates the cGAS-STING pathway through mtDNA cytoplasmic ectopia, triggering subsequent inflammatory responses (Figure 4). In agreement with this finding, abnormal mtDNA in TFAM depletion model has been reported promoting cGAS-STING-dependent IRF3 activation, which is along with increased levels of antiviral innate immune responses (West et al., 2015). However, no significant IRF3 activation was observed in TFAM mutant mice, and the STING-dependent NF-kB inflammatory signaling seems to play a promoting role in TFAM-deficiency-induced kidney failure and fibrosis. The deficiency of STING dramatically ameliorates fibrosis in mouse CKD model induced by folic acid further suggest the deleterious role of hyper-active STING signaling (Chung et al., 2019).

Variants of the apolipoprotein L1 (APOL1) gene, G1 and G2 make African-Americans prone to lupus nephritis-associated end-stage renal disease (Genovese et al., 2010). Although there is an abundance of blood-circulating nucleosome-associated double-stranded DNA fragments (nsDNA) in lupus patients (Mortensen et al., 2008), TLR9 is activated in podocytes from patients with active lupus nephritis (Machida et al., 2010). Davis et al. discovered that APOL1 expression induced by nsDNA in normal kidney podocytes involves the activation of the STING-TBK1-IRF3 pathway through transfecting nsDNA into human immortalized AB8/13 podocytes and human urine-derived MMC111.3 podocytes. In addition, cGAS cooperating with IFI16 was confirmed to be the major sensing manner of cytosolic nsDNA that mediates APOL1 expression in AB8/13 podocytes. Interestingly, blocking of IFNβ-dependent (for instance, with JAK inhibitors) and independent (with STING inhibitors) pathways could suppress APOL1 expression (Davis et al., 2019). Collectively, these results reveal that activation of the cGAS‒STING pathway facilitates kidney injury, meaning that some drugs inhibiting the pathway may be potential new therapeutic directions for preventing the progression of AKI and CKD.

### The Function of cGAS-STING Pathway in Liver Disease

Hepatocellular carcinoma (HCC), the fifth most common cancer, is the third leading cause of cancer-related death (Feng and Ben-Josef, 2011; Jemal et al., 2011). Surgical resection is an important technique in the treatment of HCC, but not all patients can undergo surgery treatment. Radiation therapy (RT) has became an important support for treatment of these patients. But radiation therapy has serious and deadly complications, such as radiation-induced liver damage (RILD). RILD, a major factor limiting radiation dose, occurs in any period of radiation therapy including a late response months or years after RT (Kim and Jung, 2017). Therefore, RILD can be divided into “classic” and “non-classic” ones. Patients with classic RILD usually have some symptoms, like fatigue, abdominal pain, increased abdominal girth, hepatomegaly, and anicteric ascites 1 to 3 months after liver radiation therapy. Additionally, the level of alkaline phosphatase (ALP) increases by more than twofold that of normal levels. The normal levels can be found for transaminase and bilirubin (Kim and Jung, 2017). Non-classic RILD patients usually have chronic hepatic diseases, such as cirrhosis and viral hepatitis. These patients have dysregulated hepatic functions with remarkably elevated serum transaminases, but not ALP (Kim and Jung, 2017). Although the RILD has been studied for many years, the understanding of its exact mechanism remains elusive. Previous studies have found that TNF-α production plays a promoting role in the development of RILD (Malik et al., 2019). In addition to the inflammatory factor of damage, mitochondrial dysfunction and oxidative stress are also important factors in inducing RILD.

**FIGURE 5:**
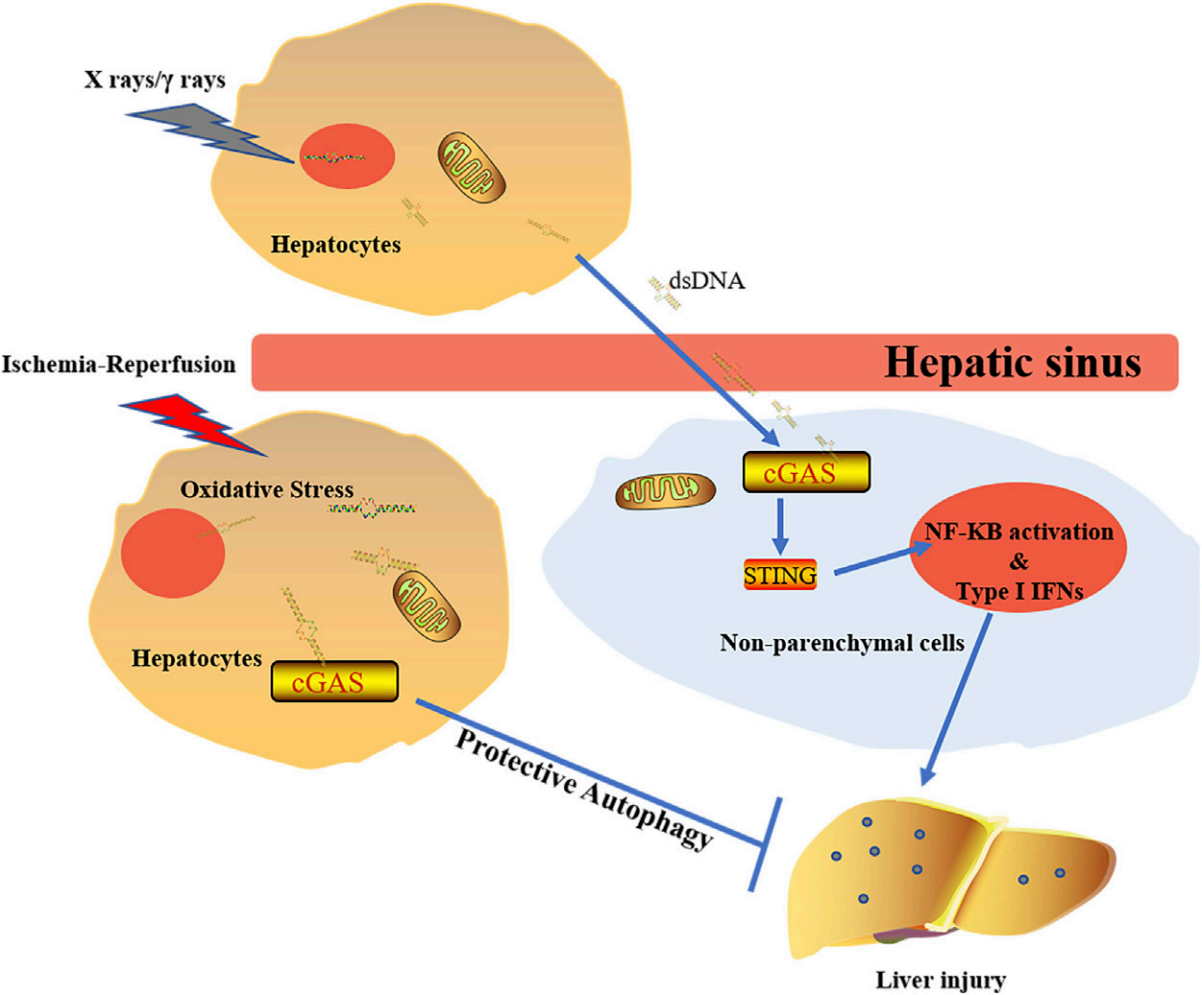
The diverse effects of cGAS doses on liver under different stimulations. The activation of cGAS-STING-IFN1 pathway induced by X rays/γ rays promotes development of RILD. Under the circumstance of ischemia-reperfusion, activated cGAS plays protective roles via regulating autophage in a STING-independent manner.

During radiation treatment on tumors, X rays and gamma rays can induce tumor cell DNA damage and mutation, leading to tumor cell death. DNA damage activates DNA sensors, triggering a subsequent inflammation. It was found that lower serum alanine aminotransferase (ALT) and aspartate aminotransferase (AST) were detected in cGAS^−/−^ mouse and STING^−/−^ mouse when compared to wild type mouse. Liver parenchyma cells under radiation release a large amount of dsDNA in the hepatic sinus. This extracellular DNA can then be identified by cGAS in non-parenchymal cells (NPCs) of the liver (Du et al., 2020). Although cGAS-STING activation increases type Ι IFNs through IRF3 and pro-inflammatory responses mediated by nuclear factor (NF)-κB (Barber, 2015), type 1 IFNs released by NPCs are the main cause of RILD in liver cells. It has been found that IFN1 has a promoting role in the development of RILD in the study of normal liver tissue adjacent to tumors of HCC patients who received RT (Figure 5) (Du et al., 2020).

The autophagic process plays an important role during degradation of damaged intracellular organelles through lysosomes. Autophagic activation can protect cells against pathological condition-induced damage (Mizushima et al., 2002; Nakai et al., 2007). Under starvation and aging conditions, autophagy can maintain cellular homeostasis to minimize tissue damage (Levine and Kroemer, 2008; Mizushima et al., 2008). Although cGAS-STING-IRF3 inflammatory reaction pathway is involved in liver injury after radiation, oxidative stress has caused many mtDNA and nuclear DNA released into the cytoplasm, leading to activation of various DNA sensors upon liver ischemia-reperfusion injury. Further studies showed that cGAS plays a protective role in liver ischemia-reperfusion injury by hepatocytes autophagy, thus reducing the cellular apoptosis and death (Figure 5). It is worth noting that the beneficial effect made by cGAS in ischemia-reperfusion injury is STING-independent (Lei et al., 2018). The decrease of cGAMP synthesis via suppressing the NTase activity of cGAS leads to amelioration of the IFN response. Activation of phosphatidylinositol 3-kinase class III induces autophagy, which facilitates the clearness of the cytosolic pathogen DNA (Liang et al., 2014). However, the concrete mechanism of cGAS regulating autophagy in ischemia-reperfusion injury is still unclear.

Viral hepatitis has long been a focus for its high morbidity and mortality. Infection of hepatitis B virus (HB-V) and hepatitis C virus (HCV) are the most common cause of death in patients with viral hepatitis. Recently, researchers found that naked relaxed-circular HBV DNA could be sensed by cGAS in hepatoma cell lines and primary human hepatocytes thereby exerting antiviral activity through activation of STING-TBK1. However, the impaired sensing during HBV infection means the occurrence of HBV evading (Verrier et al., 2018). In infected hepatocytes, the evading mechanism involves the ability of HBV to generate non-immunostimulatory RNAs or avoid sensing of its DNAs by cGAS/STING without active inhibition of the pathway (Lauterbach-Rivière et al., 2020).

### The Function of cGAS-STING Pathway in Cardiovascular Disease

In spite of rapid progress in the field of cardiovascular research and treatment strategy, the morbidity and mortality rates of ischemic heart disease are still rising, which seriously threatens human health (Kaski et al., 2018). Prolonged ischemia leads to myocardial energy metabolic disorders, falling systolic function, and ventricular remodeling, causing myocardial damage and myocardial infarction (MI) (Topol and Yadav, 2000). The MI can release numbers of DAMPs, which subsequently activate inflammatory mediators along with PRRs (Schaefer, 2014). DNA, as an important DAMPs, could be sensed by cGAS and activates cGAS-STING-IRF7 pathway during MI of mouse and human heart failure. Although the activation of this inflammatory pathway does not affect expression of major inflammatory cytokines (such as interleukin 1β, TNFα, and interleukin 6), it can govern macrophage transformation (Cao et al., 2018). M2-subtype macrophage is named as a reparative macrophage for its character of producing fibronectin (Murray et al., 2014). Compared to WT mice, myocardial repair after infarction is markedly improved in cGAS-null mice (Cao et al., 2018).

Apart from ischemia, pressure overload is also a pathogenic factor of cardiomyocyte death and mitochondrial injury (Bugger et al., 2010). Like the myocardial ischemia, cGAS-STING pathway is activated during transverse aortic constriction mouse model. It has been reported that cGAS inhibition not only ameliorates pressure overload-induced cardiac apoptosis, but also alleviates early inflammatory cell infiltration and inflammatory cytokine expression. Under different risk factors, the disparate cGAS reactions to inflammatory medium are worth our further probe into its specific mechanism.

The inflammatory factors play a critical role in cerebrovascular and neurodegenerative diseases. Not only can it appear in the moment of ischemic stroke (De Meyer et al., 2016), but also it exists in long periods after ischemia (Fu et al., 2015). Li et al. used a middle cerebral artery occlusion model to show that the cGAS-STING pathway played a certain role during stroke (Li et al., 2020). They found that A151 was able to block activation of cytosolic nucleic acid-sensing cGAS and AIM2 inflammasome (Steinhagen et al., 2018), which significantly reduced neutrophils as well as other pro-inflammatory factors to protect against brain damage via prompting neurological functional recovery and decreasing cell death.

In the model of LPS-induced cardiac dysfunction in mice, STING-NLRP3 signaling was activated. STING deficiency alleviates cardiac dysfunction, inflammation, apoptosis, and pyroptosis. LPS plays some role in the NLRP3 exportation from the nucleus to the cytoplasm by inducing ROS generation in cardiomyocytes in a STING-independent manner (Li N et al., 2019). The importance of cGAS-STING signaling has been proven that the cGAS inhibitor RU.521 accelerated the recovery of sepsis-induced cardiac dysfunction via intraperitoneal LPS (Xu et al., 2020).

### The Function of cGAS-STING Pathway in Cellular Senescence

Senescent cells are in a status of cell cycle arrest, which prevents proliferation of damaged cells along with reducing the risk of cancer. During cancer therapy, therapeutic-induced cellular senescence might be one of the main tumor suppression mechanisms (Campisi, 2013). Existing DNA damage and senescence related secretion phenotype (SASP) are key features of senescent cells. SASP is composed of various inflammatory mediators, including diversified cytokines, chemokines, extracellular matrix proteins, and growth factors (Coppé et al., 2010; Tchkonia et al., 2013). Interleukin 6 (IL6) and IL8, two key components of SASP, enhance senescent growth arrest in adjacent cells (Acosta et al., 2008; Kuilman et al., 2008).

**FIGURE 6:**
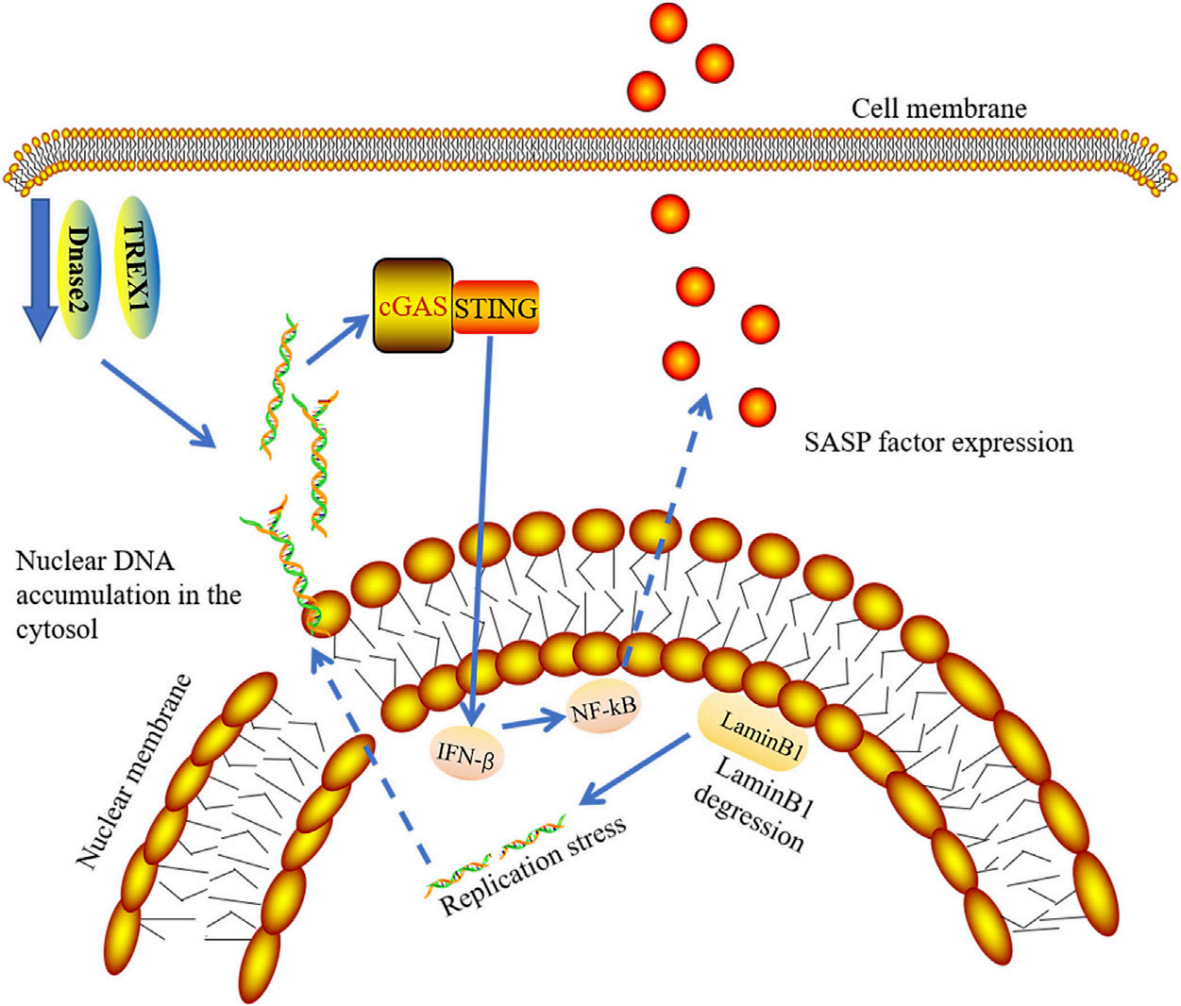
cGAS-STING signaling pathway and senescence. In senescent cells, degraded LaminB1 leads to nuclear membrane damage and chromatin fragments leaking into cytoplasm, which induces the release of SASP by activation of cGAS-STING-IFN pathway. TREX1 and Dnase2 enzymes are down-regulated in senescent cells, contributing to the accumulation of nuclear DNA in cytosol.

Although there are many stresses that induce senescence, various stress-induced DNA damage plays a major role in the induction and maintenance of senescence (Salama et al., 2014). It has been reported that the integrity of the nuclear membrane is damaged and chromatin fragments are divided into cytoplasm to become cytoplasmic chromatin fragments (CCF) due to Lamin B1 degradation in aged cells (Ivanov et al., 2013; Dou et al., 2017). Lamin B1 degradation and the emergence of CCF are vital characteristics of senescent cells (Freund et al., 2012; Ivanov et al., 2013). There is Lamin B1 degradation in senescent cells. Decreased levels of Lamin B1 results in the leakage of chromatin into the cytoplasm. Interestingly, cells with downregulation of laminin B1 have increased interferon-stimulated gene responses, which depend on cGAS sensing CCF (Figure 6). Activation of cGAS is needed to produce SASP in senescent cells (Dou et al., 2017; Glück et al., 2017; Yang et al., 2017). The absence of cGAS affects senescence induction, leading to the subsequent oncogene-activated cell clearance by immune cells (Glück et al., 2017). Existing studies have proven that DNA damage response causes abnormal accumulation of cytoplasmic DNA from the nucleus and induces abnormal activation of the cGAS-STING-IRF3 pathway along with the release of SASP (Gao et al., 2015; Gray et al., 2015; Takahashi et al., 2018). Previous studies have demonstrated the role of IFN1 in promoting aging, but the current study emphasizes the role of SASP in promoting aging *in vivo*. Therefore, it may be the combined action of several mediators that promote cGAS-dependent aging (Glück et al., 2017).

By culturing primary cells and induction of cellular senescence, Takahashi et al. showed that the expression of DNase2 and TREX1 were significantly decreased in senescence cells. They further demonstrated that the expression of both nucleases in senescence cells was downregulated at the mRNA levels. In pre-senescent cells, the decreased transcriptional activity of E2F results in accumulation of nuclear DNA in cytosol (Takahashi et al., 2018) (Figure 6).

The level of the long-interspersed element-1 (LINE-1), a retro-transposable element, was significantly increased in senescent cells (De Cecco et al., 2019). LINE-1 can transcribe mRNA to cDNA in the cytoplasm (Beck et al., 2010). Therefore, upregulation of LINE-1 in senescent cells promotes cDNA accumulation in cytosol, leading to cGAS-STING signaling activation with overproduction of SASP factors (Loo et al., 2020).

It has been reported that senescent cells secrete extracellular vesicles (like exosome) except inflammatory proteins (Takahashi et al., 2017; Takasugi et al., 2017). cGAMP can be derived from tumor cells via anti-folate transporter 1 (SLC19A1), which can activate the cGAS-STING pathway in cells (Luteijn et al., 2019; Ritchie et al., 2019). Secreting cGAMP via SLC19A1, a reverse transporter, helps cells to ingest folic acid by excreting organic phosphoric acid. This might be a new way for senescent cells to promote the paracrine immune response.

## Conclusion

Overall, the current literatures have demonstrated that cGAS-cGAMP-STING pathway play a critical role in different diseases relating to inflammation. In view of the different effects of the signaling contributing to diseases under various conditions, exacerbation or amelioration are possible. The identification of further mechanisms involved in cGAS-cGAMP-STING pathway will supply critical information on how to provide an attractive targeting therapy. Recent years have witnessed the rapid advances in the development inhibitors or agonists targeting cGAS‒STING‒TBK1. Although most drugs are used in the treatment of tumors, they can be used as reference for our treatment of other inflammatory diseases. 2′,3′ -cGAMP, as a natural CDN, conducts its anti-tumor effect via notably boosting the expression of STING and IRF3 (Li et al., 2016). Some synthetic CDNs, like ADU-S100 (ML RR-S2 CDA) (Corrales et al., 2015), a series of cAIMP analogues, (Lioux et al., 2016), are reported to dramatically activate the STING-dependent type I IFNs signaling pathways. Besides, there are other small molecule agonists specific to STING which could induce the expression of type I IFNs, including dispiro diketopiperazine (Liu B et al., 2017), benzo[b][1,4]thiazine-6-carboxamide 1 (G10), a-mangostin (Zhang et al., 2018), benzamide and its analogues (Zhang et al., 2019), dimeric amidobenzimidazoles (Ramanjulu et al., 2018), and benzothiophene derivatives. Despite pharmaceutical companies have developed numerous modulators targeting for cGAS-STING, very few drugs were qualified for clinic treatment owing to some unsolved problems involving drug-related, disease-related, and host-related obstacles (Vanpouille-Box et al., 2019).

Interaction between signaling pathways may offer some new strategies for targeted therapy. Autophagy, as an evolutionarily conserved process, is necessary to maintain cellular homeostasis for all eukaryotic cells. ABT-737, an anti-apoptotic Bcl-2 family inhibitor, can inhibit type I IFN secretion (Lindqvist et al., 2018). *In vivo* and *in vitro* experimental data showed that mammary carcinoma cells were sensitive to irradiation in case of autophagy pathway inhibition. Depletion of autophagic protein accelerates secretion of type I IFN, it can be inhibited by cGAS or STING mutation (Yamazaki et al., 2020).

Poly (ADP-ribose) polymerase (PARP), the major isoform of a family of ADP-ribosylating enzymes, has been indicated in regulating a variety of biological processes including DNA repair, gene transcription, and cell death (Sachdev et al., 2019; Eisemann and Pascal, 2020). The PARP inhibitors (e.g., Olaparib and talazoparib) are known to have promising therapeutic efficacy in BRCA-associated breast cancers (Robson et al., 2017; Litton et al., 2018). Besides, the beneficial pharmacological effects of PARP inhibitors were observed on inflammatory diseases, such as liver inflammation and fibrosis (Mukhopadhyay et al., 2014), asthma (Zaffini et al., 2018), and acute and chronic mice lung injury (Szabo et al., 2020). Recently, Pantelidou et al. revealed that interaction of PARP inhibition and STING/TBK1/IRF3 pathway activation regulates T cell recruitment and anti-tumor efficacy in cancer cells (Pantelidou et al., 2019). This achievement provides a new therapeutic pattern of targeting the cGAS-STING pathway.

Although cGAS (Bode et al., 2016) and TLR9 (Gilliet et al., 2008) mediated IFN production via sensing nucleic acids in plasmacytoid dendritic cells, they did not create synergies. Pre-stimulation of cGAS–STING in plasmacytoid dendritic cells promotes expression of SOCS1 and SOCS3, which subsequently hinders TLR9-mediated IFN production (Deb et al., 2020). This may be the protective mechanism that the body uses to prevent interferon over-expression induced autoimmune disorders.

Researchers have elaborated that Akt and AMPK play critical roles in pathological changes in the heart of obesity patients (Xu et al., 2013; Slámová et al., 2016). In Akt2-AMPK double knockout mice, inhibition of cGAS and STING could notably attenuate high fat diet induced cardiac anomalies. These results shed light on the role of Akt2-AMPK correlating with cGAS-STING signaling on autophagy/mitophagy, mitochondrial integrity, and cardiac homeostasis (Gong et al., 2020).

cGAS-cGAMP-STING mediated interferon1 signaling pathway is an important discovery in the field of natural immunity. In different tissues and organs, innate immunity plays a crucial role as the first line of defense. The study of the signal pathway will help deepen the recognition of interferon1 activation mechanisms induced by DNA virus infection, providing the theoretical foundation of drug design for autoimmune diseases and immune mediated organ damage.

## Glossary

PAMPs: pathogen-associated molecular patterns
PRRs: pattern recognition receptors
IFN: interferon
cGAS: cyclic GMP–AMP synthase
STING: stimulator of interferon genes
DAMPs: damage-related pattern molecules
TLRs: Toll-like receptors
NLRs: NOD-like receptors
CLRs: C-type lectin receptors
RLRs: RIG-I-like receptors
HMGB: high mobility group protein
mtDNA: mitochondria DNA
dsDNA: double stranded DNA
cGAMP: cyclic GMP-AMP
ssDNA: single-stranded DNA
TFAM: mitochondrial transcription factor A
CDNs: Cyclic dinucleotides
CTD: C-terminal domain
ER: endoplasmic reticulum
TBK1: TANK-binding kinase 1
NF-κB: nuclear factor κ-light-chain-enhancer of activated B cells
IRF3: interferon regulatory transcription factor 3
DsbA -L: disulfide-bond-A oxidoreductase-like protein
Banf1: Barrier-to-autointegration factor 1
MEF: mice fibroblast cells
TRIM: tripartite motif-containing protein
RNF: ring finger protein
LPS: lipopolysaccharide
TNFα: tumor necrosis factor α
GASMD: gasdermin D
BECN1: Beclin1
ENPP1: ecto-nucleotide pyrophosphatase/phosphodiesterase family member 1
SCAP: sterol regulatory element-binding protein cleavage-activating protein
NLRC3: NLR family CARD-containing 3
NETs: neutrophil extracellular traps
nDNA: nuclear DNA
ERK: extracellular regulated protein kinases
MAPK: mitogen-activated protein kinase
TPA: tissue plasminogen activator
COPD: chronic obstructive pulmonary disease
ROS: reactive oxygen species
WNT: wingless/integrase-1
PINK: PTEN-induce putative kinase
CS: cigarette smoke
AECOPD: adenovirus-associated COPD acute exacerbation
rAdVs: recombinant adenovirus vectors
NTHI: nontypeable *Hemophilus* influenzae
ILD: interstitial lung disease
ALI: acute lung injury
AKI: acute kidney injury
BAX: BCL-2-like protein 4
CKD: chronic kidney disease
VSMCs: vascular smooth muscle cells
APOL1: apolipoprotein L1
nsDNA: nucleosome-associated double-stranded DNA fragments
HCC: hepatocellular carcinoma
RT: radiation therapy
RILD: radiation-induced liver damage
ALP: alkaline phosphatase
ALT: alanine aminotransferase
AST: aspartate aminotransferase
NPCs: non-parenchymal cells
HBV: hepatitis B virus
HCV: hepatitis C virus
MI: myocardial infarction
IL: interleukin
SASP: senescence related secretion phenotype
CCF: cytoplasmic chromatin fragments
LINE-1: long-interspersed element-1
SLC19A1: anti-folate transporter 1
PARP: poly (ADP-ribose) polymerase.
